# Biogeographical distribution of bacterial communities in Changbai Mountain, Northeast China

**DOI:** 10.1002/mbo3.529

**Published:** 2018-02-15

**Authors:** Dongxue Han, Ning Wang, Xue Sun, Yanbo Hu, Fujuan Feng

**Affiliations:** ^1^ Northeast Forestry University Harbin China; ^2^ Key Lab of Wetland Ecology and Environment Northeast Institute of Geography and Agroecology Chinese Academy of Sciences Changchun China

**Keywords:** bacterial community diversity, elevation, high‐throughput sequencing, soil moisture, soil pH

## Abstract

The broad‐leaved and Korean pine mixed forest in Changbai Mountain, China is an important component of boreal forest; the area is sensitive to global climate change. To understand spatial distribution patterns of soil bacterial community along elevation, we analyzed the soil bacterial community diversity and composition along an elevational gradient of 699–1177 m in a primitive Korean pine forest in Changbai Mountain using the high‐throughput sequencing. In total, 149,519 optimized sequences were obtained. Bacterial Shannon index increased along elevation from 699 m to 937 m and started to decrease at the elevation of 1,044 m, showing a humpback curve along elevation. Evenness (ACE index) and richness (Chao index) of the soil bacterial community both decreased with elevation (the highest values of 770 and 762 at 699 m and the lowest values of 548 and 539 at 1,177 m, respectively), all the indices are significantly different between elevations. Bacterial composition at phylum and genus levels had some differences between elevations, but the dominant bacterial populations were generally consistent. Beta‐diversity analysis showed a distance‐decay pattern of bacterial community similarity at different samples. Soil physical and chemical properties explained 70.78% of the variation in bacterial community structure (soil pH explained 19.95%), and elevational distance only explained 8.42%. In conclusion, the contemporary environmental disturbances are the critical factors in maintaining the bacterial spatial distribution compared with historical contingencies.

## 1 INTRODUCTION

Soil microbe is an important component in biogeochemical cycling (Margesin, Jud, Tscherko, & Schinner, 2009; Sáenz de Miera, Arroyo, Calabuig, Falagán, & Ansola, 2014); its spatial distribution characteristics determines a level of vegetation‐soil environment interaction. Distribution pattern and functional characteristics of soil microbes have substantial impacts on growth of aboveground parts of plants (Chabrerie, Laval, Puget, Desaire, & Alard, 2003; Gömöryoá, Hrivnák, Janišová, Ujházy, & Gömöry, 2009). Therefore, studying distribution pattern of soil microbial community is very necessary to better preserve and utilize the old‐growth forests and resolve forest eco‐environment imbalances (He & Ge, 2008).

Previous researches on elevational gradient‐dependent biological diversity have mainly focused on plant and/or animal communities, but paid less attention to soil microbial community (Fierer et al., 2011; Margesin et al., 2009; Singh, Takahashi, Kim, Chun, & Adams, 2012). Research on elevation‐dependent microbial diversity is indispensable for basic ecological research and extremely crucial in forecasting responses of terrestrial ecosystems to global climate changes (Shen et al., 2014). In the past few years, modern biological techniques, particularly high‐throughput sequencing, supplied a powerful technical support for studying spatial distribution patterns of soil microbes. Recent findings showed regular changes of microbial community composition, individual abundance, or diversity with the environmental variables (Ge et al., 2008; He & Ge, 2008), such as vegetation type (Chabrerie et al., 2003; Djukic, Zehetner, Mentler, & Gerzabek, 2010; Rodríguez‐Loinaz, Onaindia, Amezaga, Mijangos, & Garbisu, 2008), soil pH (Fierer & Jackson, 2006; Männistö, Tiirola, & Häggblom, 2007; Shen et al., 2013), and spatial distance (Cho & Tiedje, 2000). However, up to date, it is still not well‐known what are the determining factors‐evolutional (e.g., geographic distance) or environmental factors (e.g., soil pH, trophic status, precipitation, and temperature) driving the soil microbial spatial distribution and how they play functions (Ge et al., 2008; He & Ge, 2008).

The Changbai Mountain is located at mid‐ to high‐latitudes, with a significant regional characteristics (Wang et al., 2015). It is very sensitive to global climate change; so has been considered as an ideal zone to study positive and negative feedback mechanisms of temperate forest to global climate change. Broad‐leaved and Korean pine mixed forest (*Pinus koraiensis* Sieb. et Zucc as an edificator) is the most representative and diverse forest ecosystem in Northeastern China (Yu et al., 2013). Recently, the broad‐leaved and primitive Korean pine forests disappeared in most of its distribution areas, whereas Changbai Mountain, as one of the core distribution areas of Korean pine forest, is still well covered by the most representative vertical gradient‐distributed old‐growth Korean pine forest (Shen et al., 2014; Yu et al., 2011, 2013). At elevations from 700 to 900 m, the main forest type is a broad‐leaved and Korean pine forest in Changbai Mountain; it turns to spruce‐fir and Korean pine mixed forests at higher elevations (Li, Bai, & Sang, 2011; Zhao, Fang, Zong, Zhu, & Shen, 2004). Here, we investigated diversity and composition of soil bacterial community and discussed underlying factors affecting vertical distribution patterns of the Korean pine forests by using Illumina High‐throughput sequencing. We hypothesized that the variation in soil physical and chemical factors and vegetation types along elevations would have important impacts on bacterial community structure. Our results will provide a reference for a better understanding of the relative contribution of soil bacterial community to the forest response to changing environmental conditions.

## 2 MATERIALS AND METHODS

### 2.1 Study area and soil sampling

The study area is located on the northern slope of Changbai Mountain Natural Reserve (41°41′49′′–42°25′18′′N, 127°42′55′′–128°16′48′′E) in Jilin Province, China. The soil type is the Haplic Cambisol (Humic, Dystric) (WRB, 2006), and the site has a typical continental temperate monsoon climate. The mean annual precipitation (MAP) increases from 800 to 1,800 mm, and mean annual temperature (MAT) decreases from 4.9 to −7.3°C with an increase in elevation. We totally collected 30 soil samples at five elevational gradients in September, 2015. The selected plots characteristics can be seen in our previous study (Ping et al., 2017). We first chose a typical primitive Korean pine forest (200 m × 400 m in area) at each elevation (699, 818, 937, 1044, 1,177 m), soil samples were collected from three plots (20 m × 20 m; about 200 m apart) as three independent replicates. In each plot, soil samples (5–10 cm and 0–5 cm depth) were collected at 10 random points (15 cm × 15 cm, composited together as a single sample), then taken back to the laboratory in bubble boxes with ice bags. The fresh soil samples were passed through a 2‐mm sieve to remove plant roots and residues, then subdivided into two subsamples: one was stored at −80°C prior to DNA extraction, another was air‐dried to measure the physical and chemical characteristics.

### 2.2 Physical and chemical properties analyses

Soil total nitrogen (TN), total organic carbon (TOC), moisture, and pH were measured according to the methods described by Ping et al. (2017).

### 2.3 DNA extraction, PCR, and sequencing

DNA was extracted from soil sample (0.25 g wet weight) using the Power Soil DNA isolation kit (MoBio Laboratories, Inc., Carlsbad, CA). Each sample was extracted and amplified for three times and then combined as one sample for high‐throughput sequencing (Liu et al., 2014). The V3 to V4 hypervariable region of bacterial 16S rRNA was amplified using the primers 338F: ACTCCTACGGGAGGCAGCA, 806R: GGACTACHVGGGTWTCTAAT. PCR reactions were performed in 20 μl system, containing 4 μl 5× FastPfu Buffer, 2 μl dNTPs (2.5 mmol/L), 0.8 μl Forward/Reverse primer (5 μmol/L), 0.4 μl FastPfu Polymerase, and 10 ng soil DNA as template; some ddH_2_O was added to the system reaching 20 μl. Thermal cycling conditions: 95°C for 3 min, followed by 27 cycles of 95° for 30 s; 55°C for 30 s; 72°C for 45 s, then an extension at 72°C for 10 min.

### 2.4 Processing and analyzing of sequencing data

Quality‐filtered and operational taxonomic units (OTUs) were clustered at 97% sequence similarity using QIIME. Chimera was detected by UCHIME. Shannon diversity index, Chao index (richness) and ACE index (evenness) were estimated at 97% sequence similarity using Mothur (Sáenz de Miera et al., 2014; Xu, Ravnskov, Larsen, Nilsson, & Nicolaisen, 2012). Beta diversity was calculated using Weighted UniFrac metric. The sequences obtained in the study have been deposited at the NCBI under BioProject ID PRJNA323515.

### 2.5 Statistical analysis

Variation partition analysis was conducted in Vegan packages in R and canonical correspondence analysis using Canoco 4.5 software. Taxonomy analysis was assigned to OTUs (at the level of 97% similarity) against Silva database, using the RDP classifier Jest algorithm in QIIME (Ping et al., 2017).

## 3 RESULTS

### 3.1 Sequence results and diversity indices

A total of 149,519 sequences were generated from the 10 soil samples. As shown in Table 1, the length of sequence varied from 434.22 bp to 440.12 bp, and the average sequence length is 437.51 bp.

**Table 1:** Miseq sequencing results and diversity estimates for soil samples

Sample	Sequencing results		Diversity estimates		
	Total sequences	Average length (bp)	Shannon	Chao	Ace
I_a_	15,729	439.38	5.72ab	770A	762A
I_b_	16,959	440.12	5.41ab	736A	728A
II_a_	13,498	439.92	5.41ab	738A	745A
II_b_	11,270	437.21	5.55ab	750A	740A
III_a_	19,039	437.63	5.86a	762A	757A
III_b_	10,843	438.51	5.77a	738A	727A
IV_a_	11,038	437.30	5.62ab	686A	685A
IV_b_	19,259	436.74	5.55ab	723A	722A
V_a_	12,021	434.22	5.11b	548B	539B
V_b_	19,863	434.49	5.18b	583B	576B

I–V: Altitude. I: 699 m; II: 818 m; III: 937 m; IV: 1044 m; V: 1177 m. a: 0–5 cm surface soil; b: 5–10 cm surface soil. The same abbreviations are used below.

Different lowercases in the same column meant significant difference at *p* = .05 among treatments; different uppercases in the same column meant significant difference at *p* = .01 among treatments.

Soil bacterial diversity (Shannon index), evenness (ACE index) and richness (Chao index) (97% sequence similarity) presented inconsistent changing patterns along elevation, and there was a significant difference between 1,177 m and others elevations (Table 1). Shannon diversity index increased along elevations from 699 to 937 m, with the highest value (5.86) at 937 m (0–5 cm surface soil); instead, it started to decrease at 1,044 m and reached the lowest value (5.11) at 1,177 m (0–5 cm surface soil). In addition, evenness index and richness index declined with elevation, with the highest values (762 and 770, respectively) at 699 m (0–5 cm surface soil), and the lowest values (539 and 548, respectively) at 1,177 m (0–5 cm surface soil).

**Figure 1:**
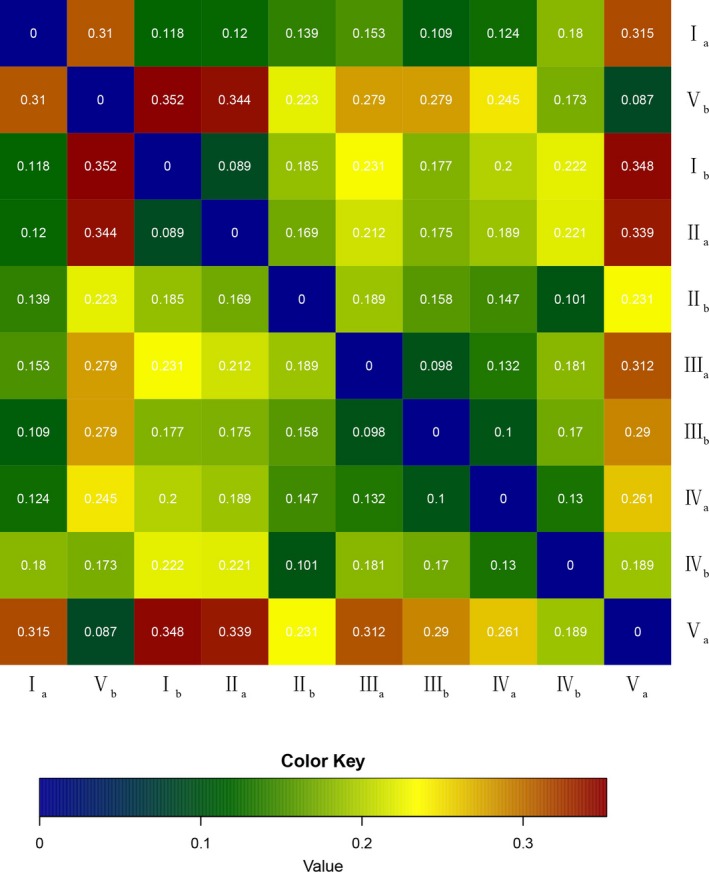
Weighted UniFrac distance for soil samples at different elevations

Beta‐diversity analysis showed that the similarity of bacterial community was significantly different between broad‐leaved forest (699–937 m) and coniferous forest (1,044–1,177 m). The similarity of bacterial communities was least between 699 m (5–10 cm surface soil) and 1,177 m (0–5 cm, 5–10 cm), the Weighted UniFrac distance was 0.348 and 0.352, respectively (Figure 1).

### 3.2 Bacterial community composition

**Figure 2:**
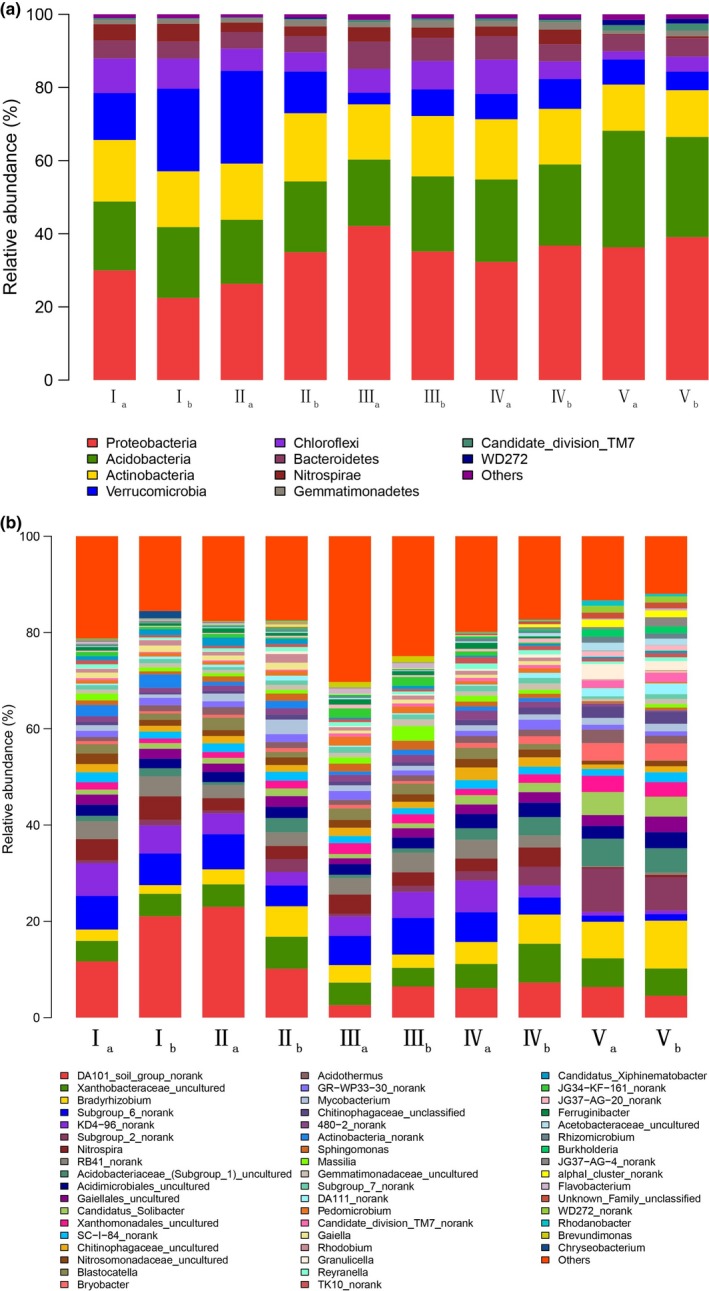
(a) Bacterial community composition at phyla level derived from the soil samples at different elevations. (b) Bacterial community composition at genera level derived from the soil samples at different elevations

At phylum level, soil bacterial community composition was consistent at different elevations (Figure 2a). A total of 23 phyla were confirmed. Among them, there were eight phyla (relative abundance over 1%) in the 17 common phyla: *Proteobacteria* (33.54%), *Acidobacteria* (21.78%), *Actinobacteria* (15.47%), *Verrucomicrobia* (10.97%), *Chloroflexi* (6.38%), *Bacteroidetes* (5.28%), *Nitrospirae* (2.88%), and *Gemmatimonadetes* (1.47%), accounting for 97.77% of the total relative abundance. *Proteobacteria*,* Acidobacteria*,* Actinobacteria,* and *Verrucomicrobia* were the most prominent phyla. The relative abundance of *Acidobacteria* exhibited a significant difference along elevations only.

**Figure 3:**
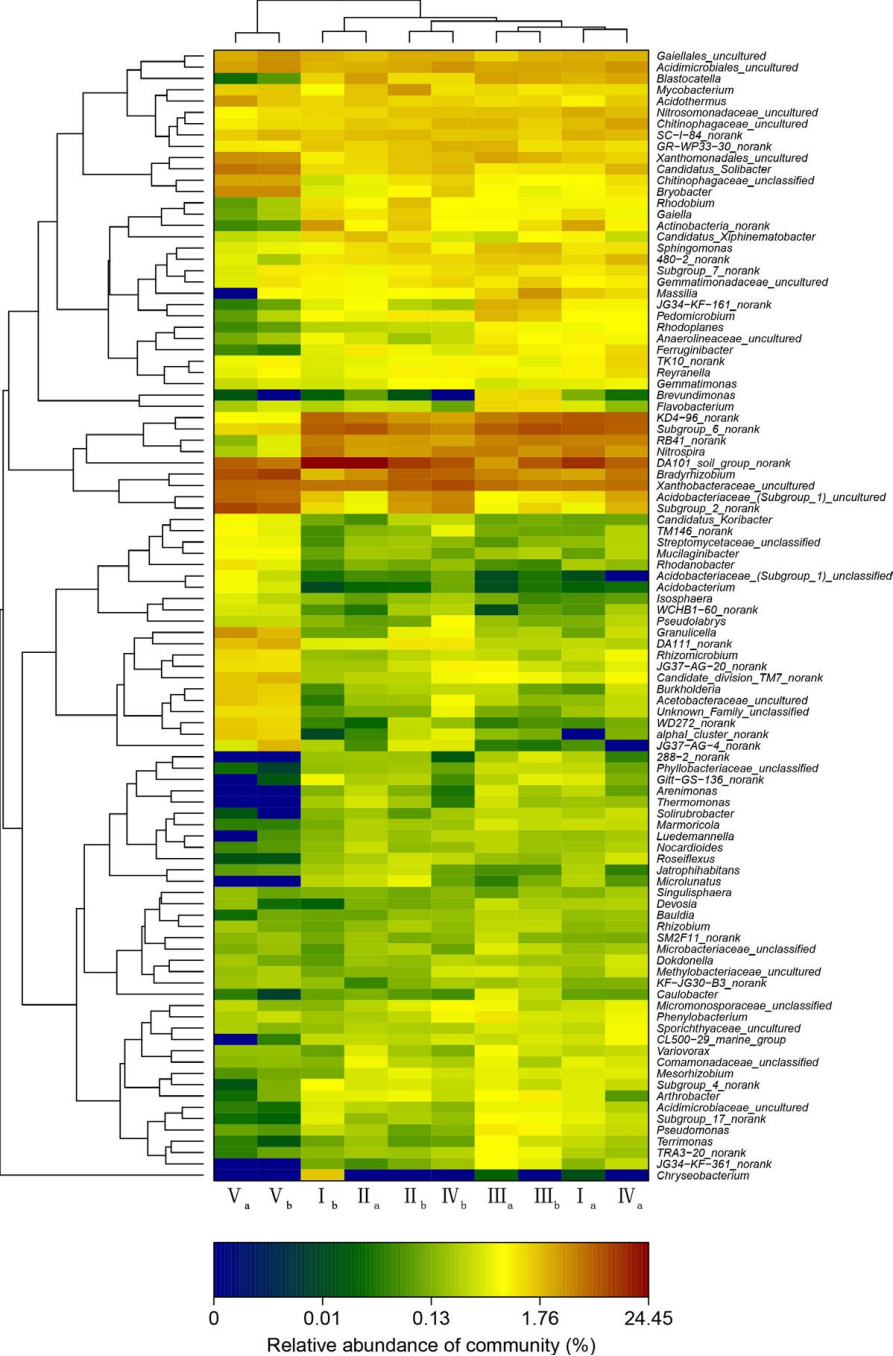
Heatmap

At the genus level, bacterial composition varied in soil samples at different elevations (Figure 2b). A total of 239 genera were identified, there were 157 common genera existed across all samples and accounted for 65.69% of total genera number and 96.48% of total relative abundance. Twenty‐six genera (relative abundance over 1%) accounted for 16.56% of common genera number and 66.02% of total relative abundance. *DA101_soil_group_norank* (9.92%), *Xanthobacteraceae_uncultured* (5.36%), *Subgroup_6_norank* (5.13%), and *Bradyrhizobium* (4.82%) were dominant genera. Relative abundance of 46 genera in the common genera was significantly different with elevation, indicating elevation′s impacts on relative abundance of the bacterial communities, particularly *Granulicella*,* WD272*,* Rhodanobacter*,* Brevundimonas*, and *KD4‐96* (Figure 3).

**Figure 4:**
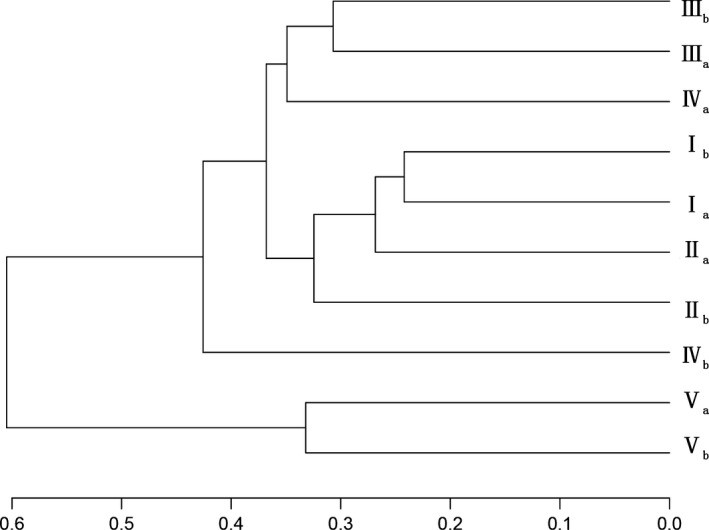
Cluster tree based on 97% similarity of community structure of soil samples

Soil bacterial communities were clustered into three groups (Figure 4): group 1 covered surface soil at 699–937 m and surface soil (0–5 cm) at 1,044 m. Group 2 was surface soil (5–10 cm) at 1,044 m. Group 3 consisted of surface soil at 1,177 m.

### 3.3 Relationship between bacterial community and environmental factors

**Figure 5:**
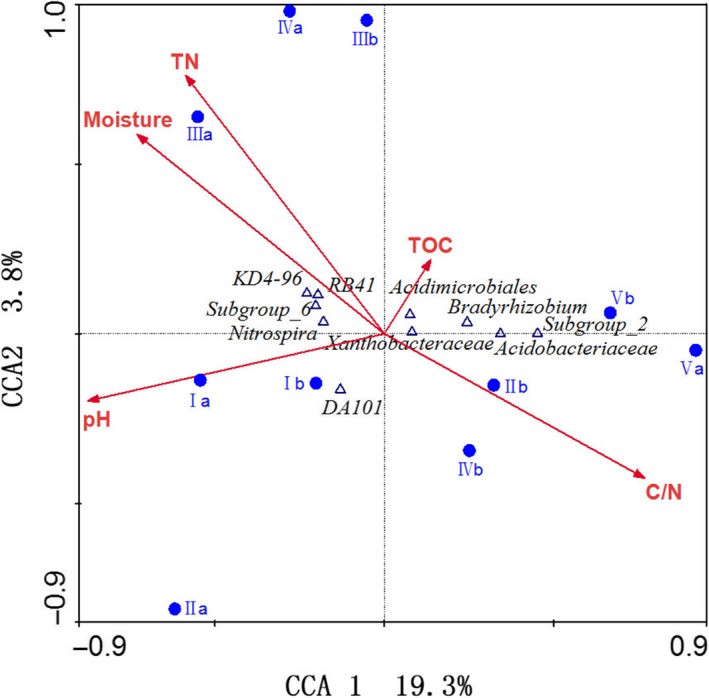
Canonical correspondence analysis (CCA) of the 10 top dominant bacterial genera, soil environmental factors and all plots

Canonical correspondence analysis suggested soil physicochemical factors significantly influenced the relative abundance of the 10 top genera (Figure 5); soil pH, C/N ratio and moisture are the main factors.

**Figure 6:**
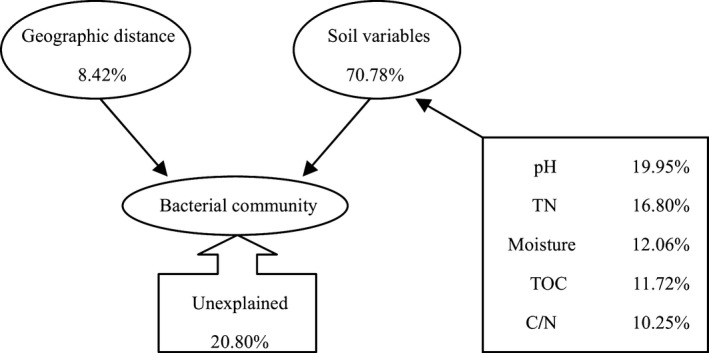
Variation partition analysis of the effects of geographic distance and soil variables on the phylogenetic structure of bacterial communities

Variation partition analysis indicated that soil physical and chemical properties explained 70.78% of the variation in soil bacterial community, whereas geographic distance (mainly elevational gradients) only explained 8.42%. Soil pH, TN, soil moisture, TOC, and C/N ratio explained 19.95%, 16.80%, 12.06%, 11.72%, and 10.25%, respectively (Figure 6).

## 4 DISCUSSION

### 4.1 Effects of elevation on bacterial diversity

Recent studies showed inconsistent conclusions on spatial distribution characteristics and diversity of soil bacterial community along elevation. Various patterns (such as monotonic decreasing, humpbacked, or hollow) along elevations were reported. For instance, Cong (2013) investigated the soil bacterial diversity of four typical vegetation types, including subalpine bush, deciduous broadleaved forest, evergreen broadleaved forest and subalpine coniferous forest, along elevations ranging from 1,000 m to 2,800 m in Shennongjia Natural Reserve using a pyrosequencing‐based metagenomic approach; they reported a pattern of monotonic decrease in soil bacterial diversity along elevation. Margesin et al. (2009) and Bryant et al. (2008) also found that bacterial diversity decreased along elevation. Singh et al. (2012) obtained a humpbacked trend in bacterial diversity along elevation from 1,000 to 3,700 m on Mount Fuji, Japan, and the diversity at 2,500 m was the highest. Wang, Soininen, He, and Shen (2012), using high‐throughput pyrosequencing, examined the bacterial community diversity and found that the distribution was hollow toward higher elevations along a gradient from 1,820 to 4,050 m on Laojun Mountain in Yunnan Province, China. While, Shen et al. (2013) and Fierer et al. (2011) observed no apparent change in soil bacterial diversity with elevation. In our study, soil bacterial diversity was increased with elevation from 699 m to 937 m and declined at the elevation of 1,044 m (Table 1), showing a humpbacked curve which was consist with the result of Singh et al. (2012). A possible explanation may lie in soil moisture, an important factor affecting bacterial diversity (Brockett, Prescott, & Grayston, 2012), and it explained 12.06% of the variation in bacterial community structure (Figure 6). In addition, aboveground vegetation composition is another factor determining soil bacterial diversity. Because vegetation types determine litter composition and soil C/N ratio, then affect microbial community structure (Djukic et al., 2010; Singh, Shi, & Adams, 2013). We discovered a gradual change in the plant composition at the elevation of 1,044 m, the presence of coniferous trees increased (Ping et al., 2017), the litter composition changed then had an impact on soil bacterial community.

In this study, we found bacterial richness and evenness declined with elevation; soil pH also declined with the increasing elevation. Soil pH explained 19.95% of the variation in bacterial community structure (Figure 6), showing a higher influence than other factors. Liu et al. (2014) and Singh et al. (2013) also suggested the soil pH had a significantly positive correlation with bacterial richness. Fierer and Jackson (2006) discovered bacterial richness and diversity were higher in neutral soils and lower in acidic soils.

### 4.2 Effects of elevation on bacterial community composition

At phylum level, there was no significant difference in soil bacterial community composition in the sample plots at different elevations. Janssen (2006) reviewed the bacterial gene banks of various soil types (tilled cropland, no‐tilled cropland, abandoned field, organic soil, mineral soil, forest, tree plantation, tilled grassland, meadow, prairie, tundra, and desert) in different regions (Italy, Norway, Germany, Russia, the United States and Netherlands). He found, among these regions and soil types, *Proteobacteria* and *Acidobacteria* were the main bacterial communities, accounting for 39% and 20%, respectively. *Actinobacteria*,* Bacteroidetes*,* Firmicutes*,* Chloroflexi*,* Gemmatimonadetes*,* Planctomycetes,* and *Verrucomicrobia* were the secondary common groups. In our study, *Proteobacteria*,* Acidobacteria*,* Actinobacteria,* and *Verrucomicrobia* were the most prominent phyla. Thus it can be concluded from the above results that at the phylum level, the dominant bacterial populations are generally consistent in different ecosystems and soil types (Janssen, 2006; Wang et al., 2014).

At the genus level, there was difference in bacterial composition at different elevations; the common genera accounted for 65.69% of total genera number and 96.48% of total relative abundance. The category of the common genera whose relative abundance exceeded 1% was consistent, whereas the relative abundance of 29.30% common genera had significant differences at different elevations. Elevation had impacts on the relative abundance of the bacterial community, particularly *Granulicella*,* WD272*,* Rhodanobacter*,* Brevundimonas*, and *KD4‐96* (Figure 3). Our result agreed with the conclusions reported by Shen et al. (2013), Zhang, Liang, He, and Zhang (2013) and Wang et al. (2014). Although the dominant bacterial species may be the same, there was considerable variation in the abundance of members in different soil types (Janssen, 2006). The abundance may be affected by soil environmental factors, including physical, chemical, and biological factors. In this article, soil pH, C/N ratio and moisture were the main factors affecting the relative abundance of prominent genera along elevation (Figure 5). Variation partition analysis demonstrated soil nutritional content, moisture, and pH had high contributions to variation in soil bacterial community with elevation (Figure 6). Furthermore, variation in plant communities was a significant factor influencing the relative abundance of bacterial communities, which was confirmed by taxonomy analysis (Figure 4).

### 4.3 Spatial distribution of bacterial community

According to the spatial distribution of soil microbial communities, two models (distance‐decay relationship and species‐area relationship) were used to describe the nonrandom distribution of microorganisms (Green & Bohannan, 2006; He & Ge, 2008). Distance–decay relationship (that is β‐diversity variation) has been a general biogeographic model reflecting microbial spatial distribution, which was adopted to investigate the change in communities composition similarity with the increasing spatial distance among samples (Green & Bohannan, 2006; Green et al., 2004). Bryant et al. (2008) reported that bacterial community was nonrandom distribution, exhibiting obvious spatial distribution with elevation, and the phylogenetic similarity decreased with distance (phylogenetic distance–decay). Martiny, Eisen, Penn, Allison, and Horner‐Devine (2011) compared the ammonia‐oxidizing bacteria community structure in 106 sediment samples from 12 salt marshes on three continents, and found the similarity between two samples declined with larger distances. In this research, β‐diversity suggested that the similarity of bacterial community among the plots declined with the increasing elevational distance, presenting a distance–decay pattern (Figure 1); bacterial community exhibited the characteristic of nonrandom distribution.

We should simultaneously consider the relative contribution of contemporary environmental factors (soil physical and chemical properties) and historical contingencies (geographic distance) when discussing the formation and maintenance mechanisms of spatial distribution pattern of the soil microbial community (Green et al., 2004; He & Ge, 2008). Variation partition analysis suggested that the impact of soil physicochemical factors on bacterial community structure was much larger than geographic distance (mainly elevational gradients) (Figure 6). Apparently, the relative contribution of contemporary environmental factors, especially soil pH, to maintaining the bacterial vertical distribution was much greater than historical factors (Liu et al., 2014; Ruamps, Nunan, & Chenu, 2011; Shen et al., 2014; Singh et al., 2013).

## 5 CONCLUSION

Elevational gradient had a significant impact on soil bacterial communities in primitive Korean pine forests in Changbai Mountain. There were significant differences in bacterial diversity, evenness and richness between elevations. Similarity of bacterial community between the sample plots decreased with the increasing vertical distance, showing a distance‐decay relationship. A possible reason may be that soil physical and chemical characteristics and aboveground plant composition varied along elevational gradients; these changes indirectly affected soil bacterial community structure. Contemporary environmental factors were the crucial factors in maintaining the bacterial vertical distribution when compared with historical factors.

## CONFLICT OF INTEREST

The authors declare that they have no conflicts of interest.

## References

[mbo3529-bib-0001] Brockett, B. F. T. , Prescott, C. E. , & Grayston, S. J. (2012). Soil moisture is the major factor influencing microbial community structure and enzyme activities across seven biogeoclimatic zones in western Canada. Soil Biology & Biochemistry, 44, 9–20.

[mbo3529-bib-0002] Bryant, J. A. , Lamanna, C. , Morlon, H. , Kerkhoff, A. J. , Enquist, B. J. , & Green, J. L. (2008). Microbes on mountainsides: Contrasting elevational patterns of bacterial and plant diversity. PNAS, 105, 11505–11511. 18695215 10.1073/pnas.0801920105 PMC2556412

[mbo3529-bib-0003] Chabrerie, O. , Laval, K. , Puget, P. , Desaire, S. , & Alard, D. (2003). Relationship between plant and soil microbial communities along a successional gradient in a chalk grassland in north‐western France. Applied Soil Ecology, 24, 43–56.

[mbo3529-bib-0004] Cho, J. C. , & Tiedje, J. M. (2000). Biogeography and degree of endemicity of fluorescent *Pseudomonas* strains in soil. Applied and Environment Microbiology, 66, 5448–5456. 10.1128/aem.66.12.5448-5456.2000 PMC92480 11097926

[mbo3529-bib-0005] Cong, J. (2013). The research of soil microbial diversity in the Shennongjia Natural Reserve. Hunan: Central South University.

[mbo3529-bib-0006] Djukic, I. , Zehetner, F. , Mentler, A. , & Gerzabek, M. H. (2010). Microbial community composition and activity in different alpine vegetation zones. Soil Biology & Biochemistry, 42, 155–161.

[mbo3529-bib-0007] Fierer, N. , & Jackson, R. B. (2006). The diversity and biogeography of soil bacterial communities. PNAS, 103, 626–631. 16407148 10.1073/pnas.0507535103 PMC1334650

[mbo3529-bib-0008] Fierer, N. , Mccain, C. M. , Meir, P. , Zimmermann, M. , Rapp, J. M. , Silman, M. R. , & Knight, R. (2011). Microbes do not follow the elevational diversity patterns of plants and animals. Ecology, 92, 797–804. 21661542 10.1890/10-1170.1

[mbo3529-bib-0009] Ge, Y. , He, J. Z. , Zhu, Y. G. , Zhang, J. B. , Xu, Z. H. , Zhang, L. M. , & Zheng, Y. M. (2008). Differences in soil bacterial diversity: Driven by contemporary disturbances or historical contingencies? ISME Journal, 2, 254–264. 18239609 10.1038/ismej.2008.2

[mbo3529-bib-0010] Gömöryoá, E. , Hrivnák, R. , Janišová, M. , Ujházy, K. , & Gömöry, D. (2009). Changes of the functional diversity of soil microbial community during the colonization of abandoned grassland by a forest. Applied Soil Ecology, 43, 191–199.

[mbo3529-bib-0011] Green, J. , & Bohannan, B. J. M. (2006). Spatial scaling of microbial biodiversity. Trends in Ecology & Evolution, 21, 501–507. 16815589 10.1016/j.tree.2006.06.012

[mbo3529-bib-0012] Green, J. L. , Holmes, A. J. , Westoby, M. , Oliver, I. , Briscoe, D. , Dangerfield, M. , … Beattie, A. J. (2004). Spatial scaling of microbial eukaryote diversity. Nature, 432, 747–750. 15592411 10.1038/nature03034

[mbo3529-bib-0013] He, J. Z. , & Ge, Y. (2008). Recent advances in soil microbial biogeography. Acta Ecologica Sinica, 28, 5571–5582.

[mbo3529-bib-0014] Janssen, P. (2006). Identifying the dominant soil bacterial taxa in libraries of 16S rRNA and 16S rRNA genes. Applied and Environment Microbiology, 72, 1719–1728. 10.1128/AEM.72.3.1719-1728.2006 PMC1393246 16517615

[mbo3529-bib-0015] Li, G. Q. , Bai, F. , & Sang, W. G. (2011). Different responses of radial growth to climate warming in *Pinus koraiensis* and *Picea jezoensis* var. komarovii at their upper elevational limits in Changbai Mountain, China. Chinese Journal of Plant Ecology, 35, 500–511.

[mbo3529-bib-0016] Liu, J. J. , Sui, Y. Y. , Yu, Z. H. , Shi, Y. , Chu, H. Y. , Jin, J. , … Wang, G. H. (2014). High throughput sequencing analysis of biogeographical distribution of bacterial communities in the black soils of northeast China. Soil Biology & Biochemistry, 70, 113–122.

[mbo3529-bib-0017] Männistö, M. K. , Tiirola, M. , & Häggblom, M. M. (2007). Bacterial communities in Arctic fields of Finnish Lapland are stable but highly pH‐dependent. FEMS Microbiology Ecology, 59, 452–465. 17328122 10.1111/j.1574-6941.2006.00232.x

[mbo3529-bib-0018] Margesin, R. , Jud, M. , Tscherko, D. , & Schinner, F. (2009). Microbial communities and activities in alpine and subalpine soils. FEMS Microbiology Ecology, 67, 208–218. 19049494 10.1111/j.1574-6941.2008.00620.x

[mbo3529-bib-0019] Martiny, J. B. , Eisen, J. A. , Penn, K. , Allison, S. D. , & Horner‐Devine, M. C. (2011). Drivers of bacterial β‐diversity depend on spatial scale. PNAS, 108, 7850–7854. 21518859 10.1073/pnas.1016308108 PMC3093525

[mbo3529-bib-0020] Ping, Y. , Han, D. X. , Wang, N. , Hu, Y. B. , Mu, L. Q. , & Feng, F. J. (2017). Vertical zonation of soil fungal community structure in a Korean pine forest on Changbai Mountain, China. World Journal of Microbiology & Biotechnology, 33, 12 https://doi.org/10.1007/s11274-016-2133-1 27885566 10.1007/s11274-016-2133-1

[mbo3529-bib-0021] Rodríguez‐Loinaz, G. , Onaindia, M. , Amezaga, I. , Mijangos, I. , & Garbisu, C. (2008). Relationship between vegetation diversity and soil functional diversity in native mixed‐oak forests. Soil Biology & Biochemistry, 40, 49–60.

[mbo3529-bib-0022] Ruamps, L. S. , Nunan, N. , & Chenu, C. (2011). Microbial biogeography at the soil pore scale. Soil Biology & Biochemistry, 43, 280–286.

[mbo3529-bib-0023] Sáenz de Miera, L. E. , Arroyo, P. , Calabuig, E. D. L. , Falagán, J. , & Ansola, G. (2014). High‐throughput sequencing of 16s RNA genes of soil bacterial communities from a naturally occurring CO_2_ gas vent. International Journal of Greenhouse Gas Control, 29, 176–184.

[mbo3529-bib-0024] Shen, C. C. , Liang, W. J. , Shi, Y. , Lin, X. G. , Zhang, H. Y. , Wu, X. , … Chu, H. Y. , (2014). Contrasting elevational diversity patterns between eukaryotic soil microbes and plants. Ecology, 95, 3190–3202.

[mbo3529-bib-0025] Shen, C. C. , Xiong, J. B. , Zhang, H. Y. , Feng, Y. Z. , Lin, X. G. , Li, X. Y. , … Chu, H. Y. , (2013). Soil pH drives the spatial distribution of bacterial communities along elevation on Changbai Mountain. Soil Biology & Biochemistry, 57, 204–211.

[mbo3529-bib-0026] Singh, D. , Shi, L. L. , & Adams, J. M. (2013). Bacterial diversity in the mountains of South‐West China: Climate dominates over soil parameters. Journal of Microbiology, 51, 439–447. 10.1007/s12275-013-2446-9 23990294

[mbo3529-bib-0027] Singh, D. , Takahashi, K. , Kim, M. , Chun, J. , & Adams, J. M. (2012). A hump‐backed trend in bacterial diversity with elevation on Mount Fuji, Japan. Microbial Ecology, 63, 429–437. 21735154 10.1007/s00248-011-9900-1

[mbo3529-bib-0028] Wang, J. , Soininen, J. , He, J. , & Shen, J. (2012). Phylogenetic clustering increases with elevation for microbes. Environmental Microbiology Reports, 4, 217–226. 23757276 10.1111/j.1758-2229.2011.00324.x

[mbo3529-bib-0029] Wang, N. N. , Wang, M. J. , Li, S. L. , Sui, X. , Han, S. J. , & Feng, F. J. (2014). Effects of variation in precipitation on the distribution of soil bacterial diversity in the primitive Korean pine and broadleaved forests. World Journal of Microbiology & Biotechnology, 30, 2975–2984. 25169822 10.1007/s11274-014-1725-x

[mbo3529-bib-0030] Wang, N. , Wang, M. J. , Li, S. L. , Wang, N. N. , Feng, F. J. , & Han, S. J. (2015). Effects of precipitation variation on growing seasonal dynamics of soil microbial biomass in broadleaved Korean pine mixed forest. Chinese Journal of Applied Ecology, 26, 1297–1305. 26571644

[mbo3529-bib-0031] WRB . (2006). World reference base for soil resources. World Soil Resources Reports, 103. Rome, FAO.

[mbo3529-bib-0032] Xu, L. H. , Ravnskov, S. , Larsen, J. , Nilsson, R. H. , & Nicolaisen, M. (2012). Soil fungal community structure along a soil health gradient in pea fields examined using deep amplicon sequencing. Soil Biology & Biochemistry, 46, 26–32.

[mbo3529-bib-0033] Yu, D. P. , Liu, J. Q. , Benard, J. L. , Zhou, L. , Zhou, W. M. , Fang, X. M. , … Dai, L. M. (2013). Spatial variation and temporal instability in the climate–growth relationship of Korean pine in the Changbai Mountain region of Northeast China. Forest Ecology and Management, 300, 96–105.

[mbo3529-bib-0034] Yu, D. P. , Wang, Q. W. , Wang, Y. , Zhou, W. M. , Ding, H. , Fang, X. M. , … Dai, L. M. (2011). Climatic effects on radial growth of major tree species on Changbai Mountain. Annals of Forest Science, 68, 921–933.

[mbo3529-bib-0035] Zhang, B. , Liang, C. , He, H. , & Zhang, X. (2013). Variations in soil microbial communities and residues along an altitude gradient on the northern slope of Changbai Mountain, China. PLoS ONE, 8, e66184. 23776630 10.1371/journal.pone.0066184 PMC3679006

[mbo3529-bib-0036] Zhao, S. Q. , Fang, J. Y. , Zong, Z. J. , Zhu, B. , & Shen, H. H. (2004). Composition, structure and species diversity of plant communities along an altitudinal gradient on the northern slope of Mt. Changbai, Northeast China. Biodiversity Science, 12, 164–173.

